# Risk factors for unplanned intensive care unit admission after esophagectomy: a retrospective cohort study of 628 patients with esophageal cancer

**DOI:** 10.3389/fonc.2024.1420446

**Published:** 2024-08-29

**Authors:** Guoqing Zhang, Shaowu Sun, Zhengxia Dong, Huang Chunyao, Zhulin Wang, Kaiyuan Li, Xu Liu, Yujie Zhang, Junya Wang, Jindong Li, Jia Zhao, Xiangnan Li

**Affiliations:** ^1^ Department of Thoracic Surgery and Lung Transplantation, The First Affiliated Hospital of Zhengzhou University, Zhengzhou, Henan, China; ^2^ Fever Clinic, The First Affiliated Hospital of Henan University of Chinese Medicine, Zhengzhou, China; ^3^ Department of Thoracic Surgery, Affiliated Hospital of Southwest Medical University, Luzhou, Sichuan, China

**Keywords:** esophageal cancer, esophagectomy, unplanned intensive care unit admission, intensive care unit, prognostic nutrition index

## Abstract

**Introduction:**

Esophagectomy patients who experience unplanned ICU admission (UIA) may experience a heavier economic burden and worse clinical outcomes than those who experience routine intensive care unit (ICU) admission. The aim of this study was to identify the risk factors for postoperative UIA in patients who underwent esophagectomy.

**Methods:**

We retrospectively included patients with esophageal cancer who underwent esophagectomy. The characteristics of postoperative UIA were described, and univariable and multivariable analyses were performed based on the logistic regression model. Furthermore, a recursive partitioning analysis was adopted to stratify the patients according to the risk of UIA.

**Results:**

A total of 628 patients were included in our final analysis, among whom 57 (9.1%) had an UIA. The patients in the UIA cohort had a higher rate of in-hospital mortality (P<0.001), longer hospital stay (P<0.001), and higher associated costs (P<0.001). Multivariable analysis showed that hybrid/open esophagectomy (OR=4.366, 95% CI=2.142 to 8.897, P<0.001), operation time (OR=1.006, 95% CI=1.002 to 1.011, P=0.007), intraoperative blood transfusion (OR=3.118, 95% CI=1.249 to 7.784, P=0.015) and the prognostic nutrition index (PNI) (OR=0.779, 95% CI=0.724 to 0.838, P<0.001) were independently associated with UIA.

**Conclusions:**

We identified several critical independent perioperative risk factors that may increase the risk of UIA following esophagectomy, and the above risk factors should be the focus of attention to reduce the incidence of postoperative UIA.

## Introduction

Esophageal cancer is the eighth most common disease in terms of incidence and the fifth most common cause of mortality (1). Esophagectomy is one of the main treatments, and routine intensive care unit (ICU) admission of patients after such high-risk surgery has often been viewed as necessary to prevent or treat life-threatening complications that could occur in the immediate postoperative period (2, 3). Most patients are not admitted to the ICU after esophagectomy routinely because of surgeon’s personal preference or limited medical resources. Thus, after esophagectomy, patients are likely to experience unplanned ICU admission (UIA) during hospitalization. However, patients who require UIA may experience a heavier economic burden and worse clinical outcomes than those who require routine ICU admission (4).

The aim of this study was to present the results of a retrospective analysis of hospitalizations to confirm the risk factors for UIA of patients who underwent esophagectomy.

## Materials and methods

### Study design and setting

In this retrospective study, we analyzed perioperative factors to identify the risk factors for postoperative UIA in patients who underwent esophagectomy. A total of 725 consecutively enrolled patients who underwent esophagectomy for esophageal cancer were screened at our center between January 2016 and July 2022. The protocol for this study was reviewed and approved by the Zhengzhou University Institutional Review Board(2022-KY-0146-002).

### Participants

**Figure 1:**
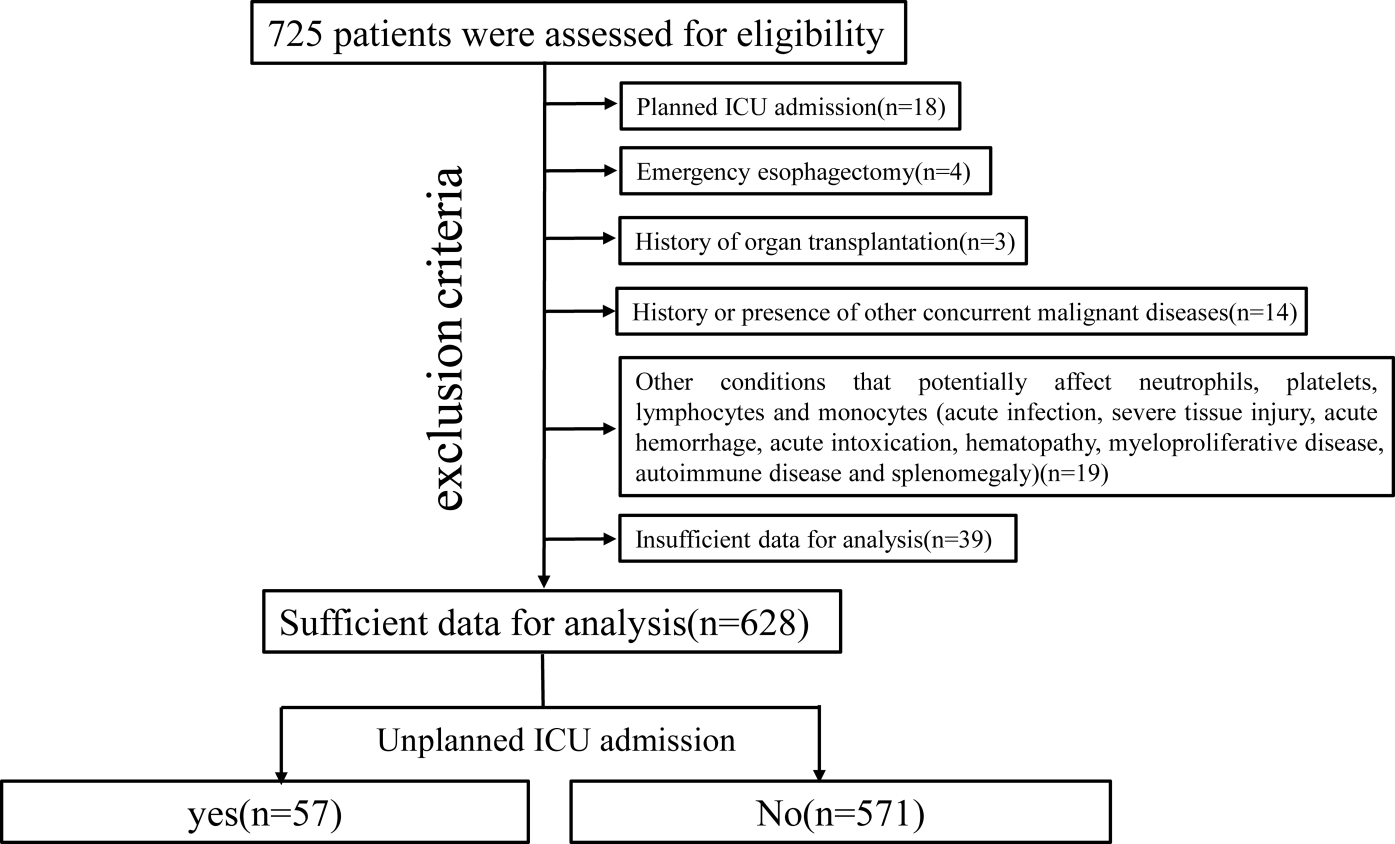
Algorithm used to identify eligible patients for this study. ICU, intensive care unit.

The criteria for planned ICU admission after esophagectomy are as follows: 1) age >80 years; 2) intraoperative hemodynamic instability; and 3) difficulty weaning from the ventilator. All patients without these conditions after esophagectomy were placed in our postoperative intensive treatment ward. Six hundred twenty-eight patients met the inclusion criteria, which are as follows: 1) histologically confirmed thoracic esophageal malignant tumor and 2) underwent radical McKeown esophagectomy (R0 resection). The exclusion criteria were as follows: 1) planned ICU admission; 2) emergency esophagectomy; 3) history of organ transplantation; 4) history or presence of other concurrent malignant diseases; 5) other conditions that potentially affect neutrophils, platelets, lymphocytes and monocytes; and 6) insufficient data for analysis (**Figure 1**).

UIA was defined as any admission to the ICU that was not anticipated pre- or intraoperatively (5). The specific indications for UIA are 1) respiratory insufficiency requiring respiratory support, 2) hemodynamic disturbance requiring hemodynamic support, or 3) an acute cardiocerebrovascular accident requiring emergency and intensive therapy.

### Surgical procedure

All included patients with thoracic ESCC underwent the McKeown procedure (thoracolaparoscopic/hybrid/open) with mediastinal and abdominal lymphadenectomy in this study cohort. After mobilization of the thoracic esophagus and abdominal stomach, a minilaparotomy incision of approximately 5 cm was made to complete gastric tube reconstruction. A linear stapler was used to create a 4 cm gastric tube. Then, the gastric tube was pulled through the posterior mediastinum and a circular stapler was used to complete the esophagogastric anastomosis. A nasogastric decompression tube and a nasojejunal feeding tube were routinely placed at the time of esophagectomy.

### Intraoperative anesthesia monitoring

We maintained a low tidal volume (5 mL/kg+5 cm H_2_O positive end expiratory pressure) during esophagectomy (6). Considering the hypotensive effect of epidural anesthesia, we did not administer epidural analgesia for esophagectomy. Intraoperative critical vital signs such as the mean arterial pressure (MAP), heart rate (HR), and PaO_2_ were recorded. Before induction of anesthesia in all patients, we inserted a 20G catheter into the radial artery with ultrasound guidance to monitor MAP. Hypotension was defined as MAP <65 mmHg for at least 1 min. Bradycardia was defined as a heart rate less than 60 beats/min or a greater than 20% decrease from the baseline heart rate. Anesthetic risk was assessed with the ASA physical status classification system (**Supplementary Table 1**) (7). Patient comorbidities were assessed with the Charlson comorbidity index (**Supplementary Table 1**) (8).

### Statistical analysis

Inflammatory biomarkers (neutrophil-lymphocyte ratio, platelet-lymphocyte ratio, lymphocyte-monocyte ratio, modified systemic inflammation score (mSIS) and prognostic nutritional index (PNI)) were tested for their ability to predict UIA (9, 10). The PNI had the strongest predictive value and was chosen for further analysis (**Supplementary Table 2**).

The primary endpoint of this study was UIA. Categorical variables were compared using the chi-square test and continuous variables were compared using Student’s t test. Variables with a P value of <0.05 in univariable analysis were included in the multivariable logistic regression model, and variables with a P value of <0.05 were regarded as statistically significant. Furthermore, a recursive partitioning analysis was adopted to stratify the patients according to the risk of UIA.

SPSS version 22 for Windows (SPSS Inc., Chicago, IL, USA) and R version 4.2.3 (https://www.r-project.org/) were used for data analysis. The data sets are available upon reasonable request.

## Results

### Patient characteristics

A total of 628 patients were included in our final analysis, among whom 57 experienced an UIA (**Figure 1**). The patients in the UIA cohort had a higher rate of respiratory system (P=0.038), cardiovascular system (P<0.001) and thromboembolic complications (P=0.003), a higher rate of in-hospital mortality (P<0.001), a longer hospital stay (P<0.001), higher associated costs (P<0.001) and worse survival (P=0.025) (**Table 1**, **Supplementary Table 3**, **Supplementary Figure 1**).

**Table 1:** Clinicopathological background of the cohorts.

Variable		Total population		
		UIA (n=57)	no UIA (n=571)	P value
**Age, mean ± SD**		64.5 ± 1.14	64.4 ± 0.32	0.931
**Sex, n (%)**	Male	42 (73.7%)	407 (71.3%)	0.701
	Female	15 (26.3%)	164 (28.7%)	
**BMI, mean ± SD**		24.2 ± 0.48	23.6 ± 0.13	0.241
**ASA classification, n (%)**	I/II	35 (61.4%)	342 (59.9%)	0.825
	III	22 (38.6%)	229 (40.1%)	
**CCI, n (%)**	0	24 (42.1%)	286 (50.1%)	0.344
	1	22 (38.6%)	202 (35.4%)	
	2	7 (12.3%)	67 (11.7%)	
	≥3	4 (7.0%)	16 (2.8%)	
**COPD, n (%)**	Yes	21 (36.8%)	140 (24.5%)	0.042
	No	36 (63.2%)	431 (75.5%)	
**Tumor location^£^, n (%)**	Upper	8 (14.0%)	72 (12.6%)	0.954
	Middle	21 (36.8%)	214 (37.5%)	
	Lower	28 (49.1%)	285 (49.9%)	
**Pathology, n (%)**	ESCC	51 (89.5%)	503 (88.1%)	0.758
	Others*	6 (10.5%)	68 (11.9%)	
**T stage^&^, n (%)**	T1	15 (26.3%)	158 (27.7%)	0.599
	T2	15 (26.3%)	180 (31.5%)	
	T3/4a	27 (47.4%)	233 (40.8%)	
**N stage^&^, n (%)**	N0	34 (59.6%)	336 (58.8%)	0.906
	N+	23 (40.4%)	235 (41.2%)	
**Neoadjuvant therapy, n (%)**	Yes	8 (14.0%)	107 (18.7%)	0.381
	No	49 (86.0%)	464 (81.3%)	
**McKeown procedures, n (%)**	Minimally invasive	28 (49.1%)	493 (86.3%)	<0.001
	Hybrid/open^#^	29 (50.9%)	78 (13.7%)	
**Lymphadenectomy, n (%)**	Two-field	48 (84.2%)	492 (91.1%)	0.685
	Three-field	9 (15.8%)	79 (13.8%)	
**Intraoperative hypotension, n (%)**	Yes	8 (14.0%)	103 (18.0%)	0.450
	No	49 (86.0%)	468 (82.0%)	
**Intraoperative bradycardia, n (%)**	Yes	22 (38.6%)	263 (46.1%)	0.281
	No	35 (61.4%)	308 (53.9%)	
**Operation time, mean ± SD**		344.1 ± 9.98	306.1 ± 2.90	<0.001
**Blood transfusion, n (%)**	Yes	17 (29.8%)	26 (4.6%)	<0.001
	No	40 (70.2%)	545 (95.4%)	
**RLNP, n (%)**	Yes	4 (7.0%)	54 (9.5%)	0.544
	No	53 (93.0%)	517 (90.5%)	
**PNI**		46.7 ± 0.85	54.3 ± 0.21	<0.001

ASA, American Society of Anesthesiologists; BMI, body mass index; CCI, Charlson Comorbidity Index; COPD, chronic obstructive pulmonary disease; ESCC: esophageal squamous cell carcinoma; N, node; PNI, prognostic nutrition index; RLNP, recurrent laryngeal nerve paralysis; SD, standard deviation; T, tumor; UIA, unplanned intensive care unit admission.

*Adenocarcinoma and small cell carcinoma.

Including thoracotomy and/or laparotomy.

^&^Clinical stage at first diagnosis on the basis of chest CT and ultrasound gastroscopy; T3 and T4a stages were combined in the analysis.

**^£^** The tumor location was categorized according to the 12th edition of the Japanese Classification of Esophageal Cancer.

**Figure 2:**
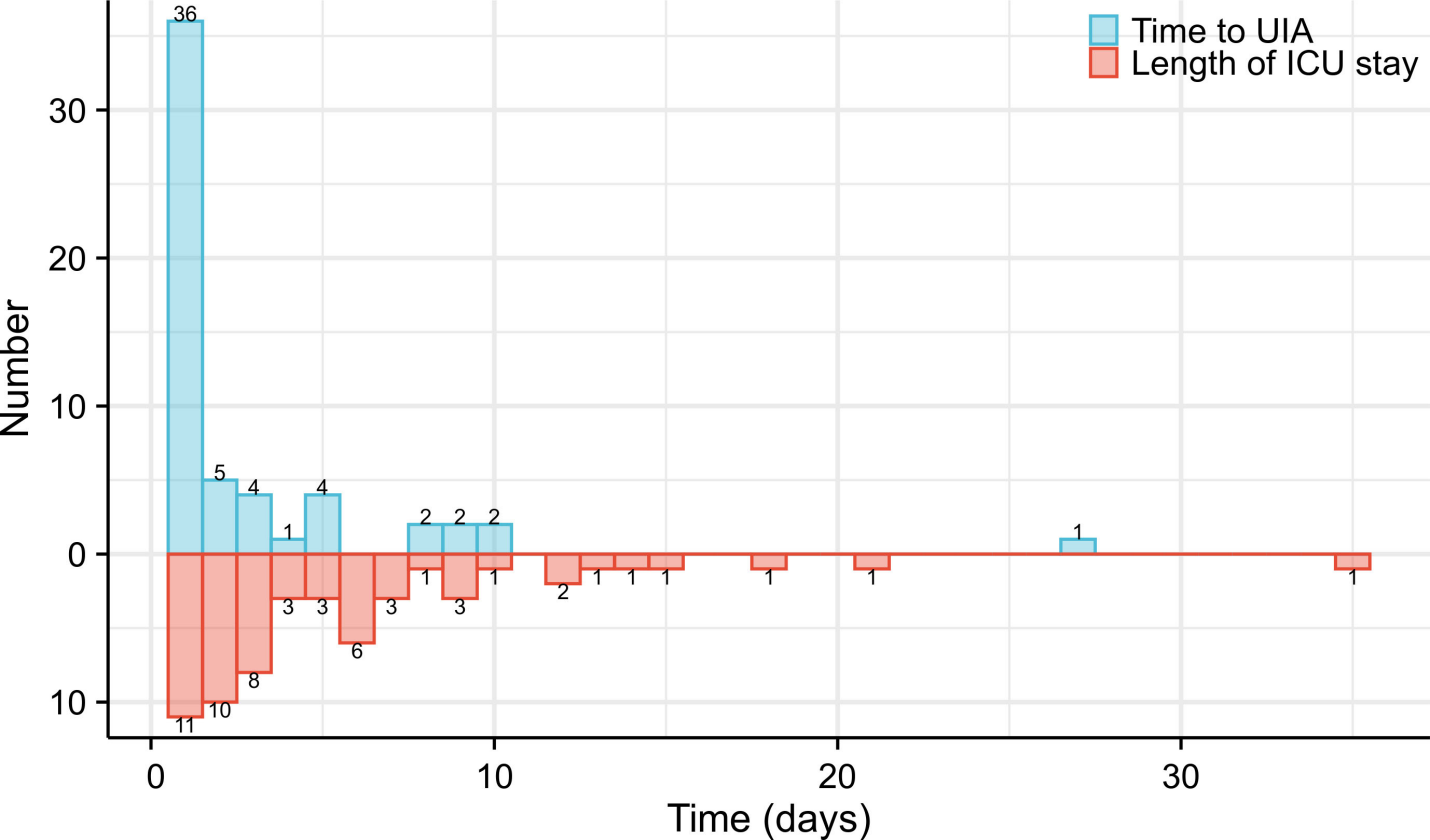
Time to UIA after esophagectomy and Length of stay in the ICU after unplanned admission.

A total of 78.9% of patients required UIA within 3 days, and the median time to UIA was 1 postoperative day (1,27). The patient with the latest UIA occurrence (27 days after esophagectomy) experienced respiratory failure induced by an anastomotic fistula (**Figure 2**). The median length of ICU stay after UIA was 3 (1, 35) days, and the longest stay was 35 days (**Figure 2**). 52.6% (30/57) of the patients were reintubated, and the median tracheal intubation time was 4 days. The median ICU stay was 4 days. Of note, the reasons for UIA were mostly related to respiratory conditions during their postoperative course in general wards. Of the 57 patients who had an UIA, 43 (75.4%) suffered from respiratory failure, and 16 (28.1%) suffered from cardiovascular conditions. As one of the main postoperative complications, anastomotic fistula was thought to have caused respiratory failure or septic shock in 15 (26.3%) patients. Additionally, 2 (3.5%) patients were confirmed to have incomplete intraoperative hemostasis (intraoperative iatrogenic errors) and were admitted to the ICU after an unplanned second operation to stop the bleeding (**Table 2**).

**Table 2:** Characteristics of postoperative UIA (N= 57).

Variable		
**Time to UIA**	POD, median(min, max)	1(1, 27)
**Reintubation**	n(%)	30(52.6%)
**Tracheal intubation time**	Median(min, max)	4(1, 22)
**Median length of ICU stay**	Median(min, max)	3(1, 35)
Reasons for UIA*		
Respiratory system	Respiratory failure, n(%)^$^	43(75.4%)
Cardiovascular system	Cardiac failure, n(%)	3(5.3%)
	Hypotensive shock, n(%)	9(15.8%)
	Hypertensive crisis, n(%)	1(1.8%)
	Arrhythmia, n(%)	3(5.3%)
Acute cardiocerebrovascular accident	Pulmonary embolism, n(%)	2(3.5%)
	Myocardial infarction, n(%)	4(7.0%)
	Cerebral infarction, n(%)	3(5.3%)
Anastomotic fistula	Causing respiratory failure or septic shock, n(%)	15(26.3%)
Iatrogenic errors^#^	Incomplete intraoperative hemostasis, n(%)	2(3.5%)
Others	Septic shock, n(%)	5(8.8%)
Direct reasons for UIA*	Respiratory failure, n(%)	43(75.4%)
	Hypotensive shock, n(%)	9(15.8%)
	Disturbance of consciousness, n(%)	26(45.6%)
**Destination after ICU discharge**	Department of thoracic surgery, n(%)	47(82.5%)
	ICU discharge, n(%)	9(15.8%)
	Department of Oncology, n(%)	1(1.8%)

ICU, intensive care unit; POD, postoperative day; UIA, unplanned intensive care unit admission.

*One who experienced UIA for two or more reasons.

^$^The causes of respiratory failure were pulmonary atelectasis (sputum obstructing the airway, massive pleural effusion) and pulmonary inflammation; respiratory failure secondary to cardiovascular or cardiocerebrovascular accidents is listed separately.

^#^Admitted to the ICU after an unplanned second operation to stop bleeding.

### Risk factors for UIA

The overall clinicopathological characteristics for the included cohort are summarized in **Table 1**, which shows that the patients requiring an UIA were significantly more likely to be diagnosed with COPD (P=0.042), to have undergone a hybrid/open procedure (P=0.002), to have a longer operation time (P<0.001), to receive an intraoperative blood transfusion (P<0.001) and to have a lower PNI (P<0.001). Furthermore, as shown in **Table 3**, univariable logistic regression analysis revealed that being diagnosed with COPD (OR=1.796, 95% CI=1.015 to 3.179, P=0.044), undergoing a hybrid/open procedure (OR=6.546, 95% CI=3.696 to 11.594, P<0.001), having a longer operation time (OR=1.007, 95% CI=1.003 to 1.011, P<0.001), and being more likely to receive an intraoperative blood transfusion (OR=8.909, 95% CI=4.466 to 17.770, P<0.001) were predictors for UIA. A higher preoperative PNI (OR=0.749, 95% CI=0.699 to 0.803, P<0.001) was a protective factor for UIA. Also, we conducted a subgroup analysis comparing minimally invasive versus hybrid/open procedures, and the results demonstrated that patient underwent hybrid/open esophagectomy was more likely to receive an intraoperative blood transfusion (**Supplementary Table 4**).

**Table 3:** Univariable logistic regression analysis of the risk factors for UIA.

Variable		Univariable analysis	
		OR (95% CI)	P value
**Age, mean ± SD**		1.002 (0.967-1.038)	0.931
**Sex, n (%)**	Male	baseline	
	Female	0.886 (0.478-1.642)	0.701
**BMI, mean ± SD**		1.054 (0.965-1.151)	0.241
**ASA classification, n (%)**	I/II	baseline	
	III	0.939 (0.537-1.642)	0.825
**CCI, n (%)**	0	baseline	0.320
	1	1.298 (0.708-2.379)	0.399
	2	1.245 (0.515-3.011)	0.627
	≥3	2.979 (0.923-9.620)	0.068
**COPD, n (%)**	Yes	1.796 (1.015-3.179)	0.044
	No	baseline	
**Tumor location, n (%)**	Upper	baseline	0.954
	Middle	0.883 (0.375-2.081)	0.776
	Lower	0.884 (0.387-2.022)	0.771
**Pathology, n (%)**	ESCC	baseline	
	Others*	0.870 (0.360-2.104)	0.758
**T stage, n (%)**	T1	baseline	0.600
	T2	0.878 (0.416-1.852)	0.732
	T3	1.221 (0.629-2.368)	0.555
**N stage, n (%)**	N0	baseline	
	N+	0.967 (0.555-1.684)	0.906
**Neoadjuvant therapy, n (%)**	Yes	baseline	
	No	1.412 (0.650-3.070)	0.383
**McKeown procedure, n (%)**	Minimally invasive	baseline	
	Hybrid/open^#^	6.546 (3.696-11.594)	<0.001
**Lymphadenectomy, n (%)**	Two-field	baseline	
	Three-field	1.168 (0.551-2.473)	0.686
**Intraoperative hypotension, n (%)**	Yes	baseline	
	No	1.348 (0.620-2.933)	0.451
**Intraoperative bradycardia, n (%)**	Yes	baseline	
	No	1.358 (0.777-2.374)	0.282
**Operation duration, mean ± SD**		1.007 (1.003-1.011)	<0.001
**Blood transfusion, n (%)**	Yes	8.909 (4.466-17.770)	<0.001
	No	baseline	
**RLNP, n (%)**	Yes	baseline	
	No	1.384 (0.482-3.972)	0.546
**PNI**		0.749 (0.699-0.803)	<0.001

ASA, American Society of Anesthesiologists; BMI, body mass index; CCI, Charlson Comorbidity Index; CI, confidence interval; COPD, chronic obstructive pulmonary disease; ESCC: esophageal squamous cell carcinoma; N, node; OR, odds ratio; PNI, prognostic nutrition index; RLNP, recurrent laryngeal nerve paralysis; SD, standard deviation; T, tumor; UIA, unplanned intensive care unit admission.

Including thoracotomy and/or laparotomy.

**Figure 3:**
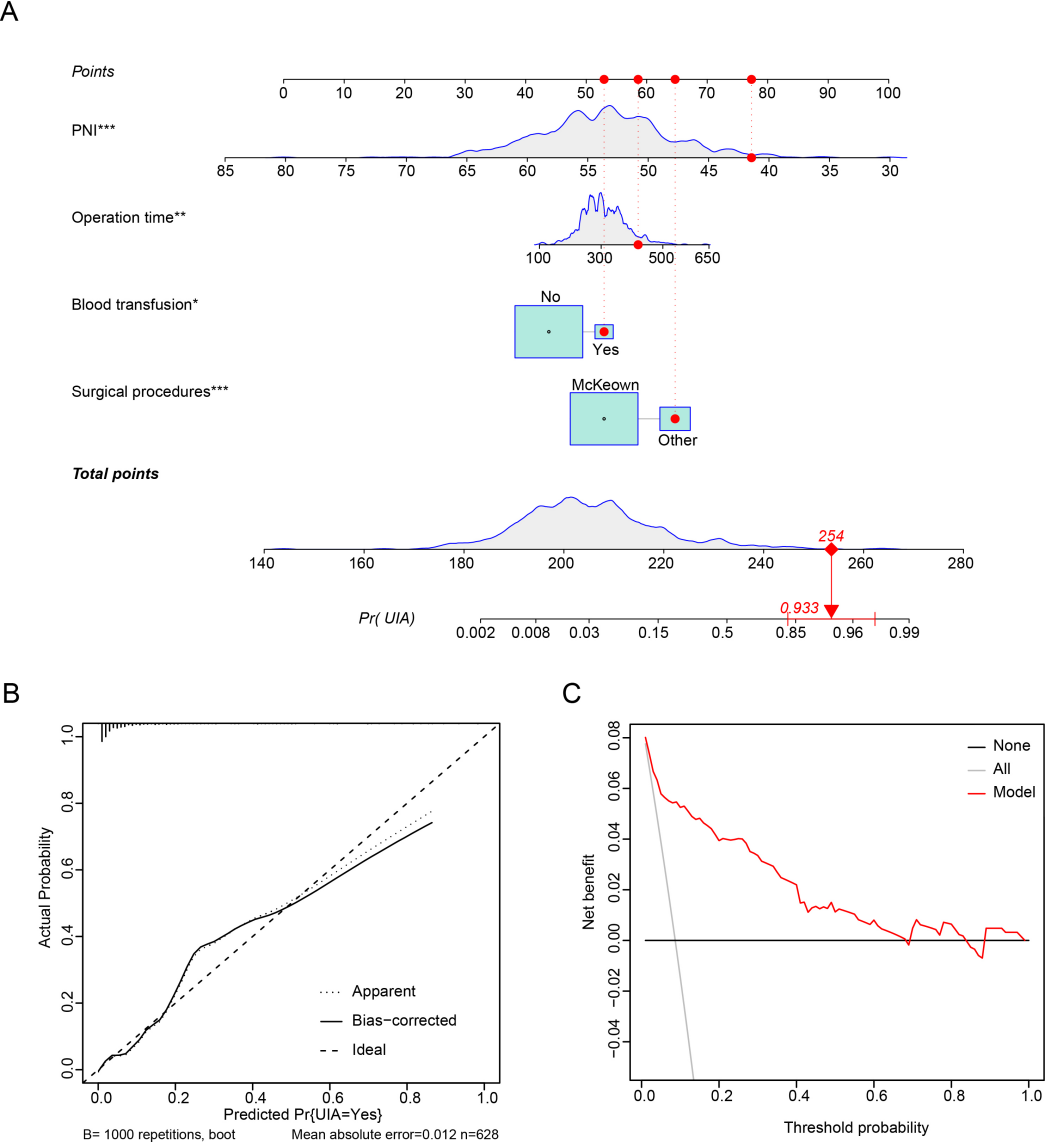
Prediction model for predicting UIA of patients who underwent esophagectomy. **(A)** Nomogram. **(B)** Calibration plots. **(C)** Decision curve analysis.

**Figure 4:**
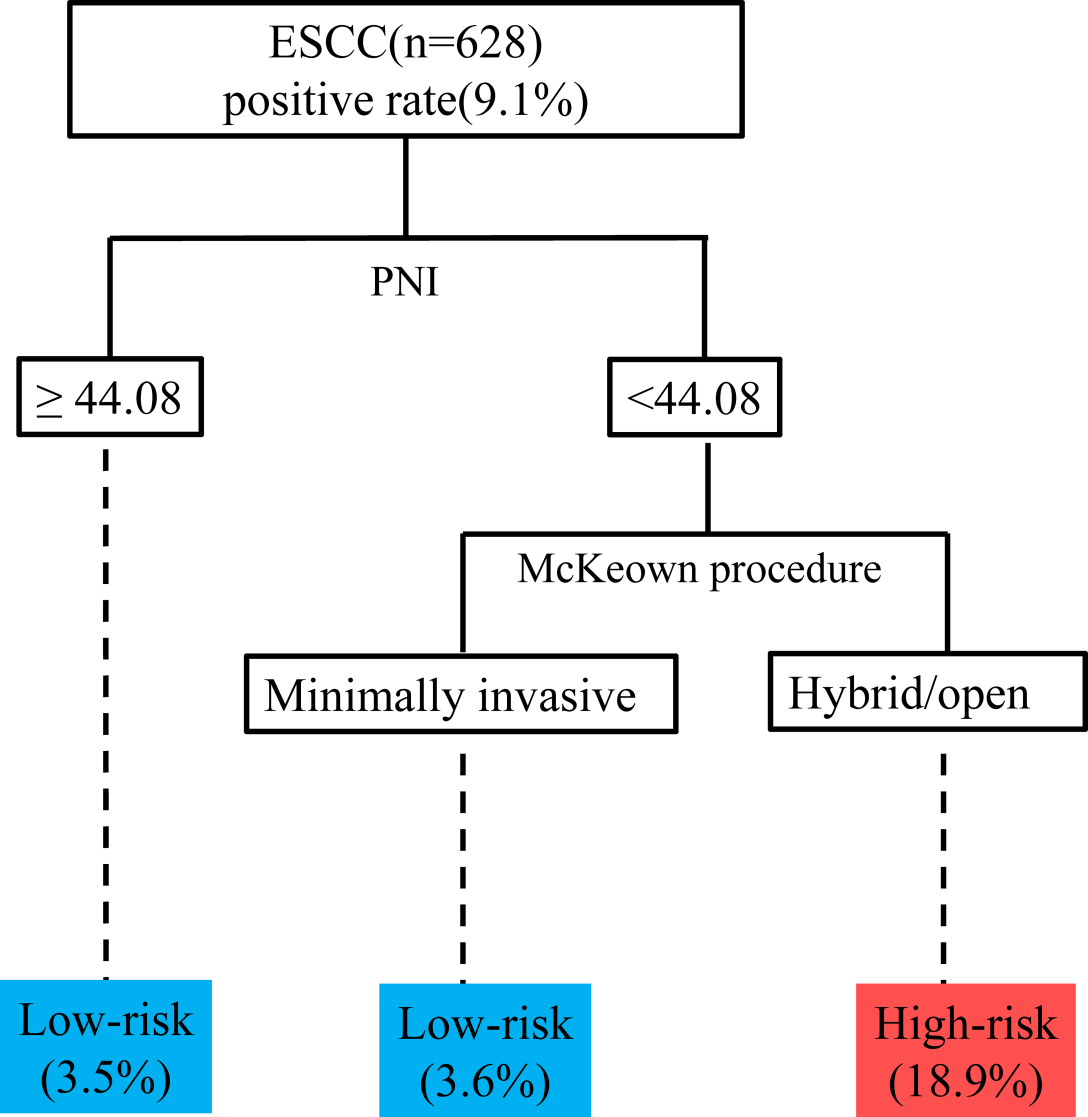
Recursive partitioning analysis for risk stratification according to the probability of UIA. ESCC, esophageal squamous cell carcinoma; PNI, prognostic nutrition index; UIA, unplanned intensive care unit admission.

We then included variables with a P value of <0.05 in the multivariable model (potential interactions were explored); hybrid/open esophagectomy (OR=4.290, 95% CI=2.085 to 8.786, P<0.001), operation time (OR=1.049, 95% CI=1.011 to 1.090, P=0.012), intraoperative blood transfusion (OR=2.964, 95% CI=1.201 to 7.315, P=0.018) and PNI (OR =0.524, 95% CI=0.391 to 0.701, P<0.001) were independently associated with UIA (**Table 4**). The prediction model is graphically presented as a nomogram (**Figure 3A**), and calibration curves showed that the UIA probabilities predicted by the nomogram were well matched with the actual probabilities (**Figure 3B**). Decision curve analysis yielded a range of threshold probabilities (0.001–0.835) at which the clinical net benefit of the risk model was greater than that in hypothetical all-screening or no-screening scenarios (**Figure 3C**). Furthermore, the patients were divided into 2 risk groups based on the PNI and the McKeown procedure: (I) the low-risk group: patients with a PNI ≥44.08, patients with a PNI <44.08 and who underwent minimally invasive procedures, and (II) the high-risk group: patients with a PNI <44.08 and who underwent hybrid/open procedures. The risk of UIA was 3.5-3.6% for the low-risk group and 18.9% for the high-risk group (**Figure 4**).

**Table 4:** Multivariable logistic regression analysis of the risk factors for UIA.

		OR (95% CI) and P value	
		Multivariable Adjustment	
**COPD**		0.876 (0.420-1.825)	0.723
**McKeown procedures**	Minimally invasive	baseline	
	Hybrid/open^#^	4.290 (2.085-8.786)	<0.001
**Operation duration**		1.049 (1.011-1.090)	0.012
**Blood transfusion**		2.964(1.201-7.315)	0.018
**PNI**		0.524 (0.391-0.701)	<0.001
PNI * Operation duration		0.999(0.998-1.000)	0.004

CI, confidence interval; COPD, chronic obstructive pulmonary disease; OR, odds ratio; PNI, prognostic nutrition index; UIA, unplanned intensive care unit admission.

^#^Including thoracotomy and/or laparotomy.

## Discussion

In this study, 9.1% of patients who underwent esophagectomy required unplanned postoperative ICU admission, and preoperative indicators (lower PNI) and intraoperative indicators (hybrid/open esophagectomy, longer operation time and intraoperative blood transfusion) were independent risk factors for UIA. Thus, targeted strategies for reducing the influence of perioperative risk factors to avoid ICU admission have important clinical significance. The incidence of UIA can potentially be reduced by active preoperative intervention and operation improvement.

First, we investigated the effects on UIA of operational procedures and the results indicated that hybrid minimally invasive esophagectomy/open esophagectomy was associated with a significantly higher incidence of UIA than minimally invasive esophagectomy was. With the development of minimally invasive techniques, minimally invasive esophagectomy has accelerated the rate of postoperative recovery (11), reduced the incidence of postoperative complications and led to a better quality of life for patients compared to open esophagectomy (12–15). In addition to the surgeon’s preferences, patients who undergo hybrid minimally invasive esophagectomy/open esophagectomy may have more complex conditions, such as larger tumors, more severe pleural or abdominal cavity adhesions, and massive intraoperative bleeding. These factors not only increase the difficulty of esophagectomy but also prolong the operation time, which further increases the incidence of UIA.

Second, we noted that patients who received an intraoperative transfusion had a higher incidence of UIA, with an OR=2.964. Esophagectomy is an extensive and complex procedure, and the transfusion rate ranges from 7.3% to 80.8% (16). In our study, the transfusion rate was 29.8% in the UIA cohort and 4.6% in the control group. Melis et al. demonstrated that transfusion was significantly associated with postoperative overall complications (17). However, no study has explored the effect of transfusion on the occurrence of UIA. In our study, we note that patients who receive an intraoperative transfusion are more likely to experience UIA. The underlying mechanisms for these associations are not completely understood. Allogenic transfusion has been confirmed to suppress the innate and acquired immune systems, which may be associated with tumor recurrence in patients with esophageal cancer (18). In addition, patients who receive transfusions are known to have preoperative anemia, be older and have a significant systemic inflammatory response secondary to esophagectomy, which may further contribute to and exacerbate postoperative complications.

Third, we found that the PNI was an independent predictor of UIA. Furthermore, a low PNI has been shown to be associated with advanced age, higher tumor stage, and lymph node metastases (9). However, how can we explain the ability of the PNI to predict perioperative complications? The underlying molecular mechanism may be associated with immune conditions. Studies have shown that low lymphocyte counts may indicate an increased susceptibility to infection (19). Additionally, major surgeries can induce a marked shift in the Th1/Th2 balance toward Th2, which will causes immunosuppression and increased susceptibility to postoperative infection (20, 21). Thus, a low PNI in patients who undergo esophagectomy may facilitate immunosuppression and indicate malnutrition, thus leading to UIA.

The implications of this study for clinical practice are as follows. 1. Patients had an UIA mainly for respiratory complications, and the median time to UIA after esophagectomy was 1 day. The potential factors were the preoperative inflammatory state, poor quality of airway management, insufficient intraoperative lung protection and long operation time. To offset these intraoperative disadvantages, a more aggressive approach is needed to control pneumonia before surgery, protect the airway during surgery, and control the condition of the lungs (bedside bronchoscopy for sputum aspiration, etc.) after surgery. 2. Intraoperative factors such as hybrid/open esophagectomy, a longer operation time and intraoperative blood transfusion are critical for postoperative UIA. 3. A lower PNI can serve as a warning to clinicians to carefully assess a patient’s clinical condition and possibly adjust the patient’s intervention. 4. ERAS protocols for esophagectomy should be implemented on a case-by-case basis. ERAS protocols such as early extubation should not be aggressively pursued for high-risk patients. The risk factor model should be used to guide clinical interventions and in combination with current clinical practice to more accurately identify patients who require UIA postoperatively. More importantly, aggressive interventions targeting risk factors might prevent an UIA. Further research is needed to verify the effectiveness of these interventions.

The major limitations of our study are its retrospective, single-center design and a considerable amount of missing data (97 patients were excluded from the analysis, 39 had insufficient data). Second, we did not consider sufficient factors that can potentially influence the incidence of UIA (22), such as intraoperative anesthesia management data (such as fluid management (23), ventilator settings (6)), diabetes mellitus (24), chronic kidney disease (25) and long-term drug use (26). It is possible that the lack of such information led to bias in the conclusion. Third, few patients undergo neoadjuvant therapy because high-quality multidisciplinary treatment (MDT) is not routinely administered in the early stage, which may also bias our conclusion. Forth, some patients with deteriorated conditions may not be transferred to the ICU for economic reasons, which can also lead to biased results. Finally, there is a potential for ‘delayed’ or ‘less qualified’ responses to complex patients outside the ICU, which may also confer bias to the conclusion.

## Conclusion

We identified several critical independent risk factors that may indicate an increased risk of UIA following esophagectomy. Our results showed that it is pivotal to reduce the incidence of UIA through sophisticated intraoperative manipulation and perioperative management. Large-scale prospective research is needed to verify our findings.

## Data Availability

The original contributions presented in the study are included in the article/**Supplementary Material**. Further inquiries can be directed to the corresponding authors.
