# Supplementary material for: Risk factors for unplanned intensive care unit admission after esophagectomy: a retrospective cohort study of 628 patients with esophageal cancer
Source: Front Oncol. 2024 Aug 29;14:1420446. doi: 10.3389/fonc.2024.1420446 (PMC11390390; doi:10.3389/fonc.2024.1420446)
Supplement: Supplementary file 1 [file Table1.docx]

**Supplementary Table 1. ASA physical status classification system and Charlson Comorbidity Index**

| **ASA physical status classification system** | | |
| --- | --- | --- |
| **ASA-physical status class** | **definition** | **Examples, including but not limited to** |
| I | A normal patient | Healthy, nonsmoking, no or minimal alcohol use |
| II | A patient with mild systemic disease | Mild diseases only without substantive functional limitations. Examples include(but not limited to) current smoker, social alcohol drinker, pregnancy, obesity(30<BMI<40), well-controlled DM/HTN, mild lung disease |
| III | A patient with severe systemic disease | Substantive functional limitations; one or more moderate to severe diseases. Examples include(but not limited to) poorly controlled DM or HTN, COPD, morbid obesity (BMI≥40), active hepatitis, alcohol dependence or abuse, implanted pacemaker, moderate reduction of ejection fraction, ESRD requiring regularly scheduled dialysis, premature infant PCA<60 weeks, history(>3 months) of MI, CVA, TIA, OR CAD/stents |
| IV | A patient with severe systemic disease that is potentially fatal | Examples include(but not limited to) recent(<3 months) MI, CVA, TIA, OR CAD/stents, ongoing cardiac ischemia or severe valve dysfunction, severe reduction of the ejection fraction, sepsis, DIC, ARDS, or ESRD not requiring regularly scheduled dialysis |
| V | A moribund patient who is not expected to survive without the operation | Examples include(but not limited to) ruptured abdominal/thoracic aneurysm, massive trauma, intracranial bleed with a mass effect, bowel ischemia accompanied by significant cardiac pathology or multiple organ/system dysfunction |
| VI | A brain-dead patient whose organs are being removed for donor purposes |  |
| **Charlson Comorbidity Index** | | |
|  | **Assigned weights for diseases** | **Conditions** |
|  | 1 | Myocardial infarct; Congestive heart failure; Peripheral vascular disease; Cerebrovascular disease; Dementia; Chronic pulmonary disease; Connective tissue disease; Ulcer disease; Mild liver disease; Diabetes; |
|  | 2 | Hemiplegia; Moderate or severe renal disease; Diabetes with end organ damage; Any tumor; Leukemia; Lymphoma; |
|  | 3 | Moderate or severe liver disease; |
|  | 6 | Metastatic solid tumor; AIDS; |
