# Supplementary material for: Risk factors for unplanned intensive care unit admission after esophagectomy: a retrospective cohort study of 628 patients with esophageal cancer
Source: Front Oncol. 2024 Aug 29;14:1420446. doi: 10.3389/fonc.2024.1420446 (PMC11390390; doi:10.3389/fonc.2024.1420446)
Supplement: Supplementary file 2 [file Table2.docx]

**Supplementary Table 2. Univariable analysis of measures of inflammation in patients who experienced UIA.**

| **Inflammatory biomarkers** | | **OR (95% CI) and P value** | |
| --- | --- | --- | --- |
|  |  | Univariable analysis | |
| **NLR** |  | 1004 (0.972-1.038) | 0.798 |
| **PLR** |  | 0.999 (0.995-1.004) | 0.790 |
| **LMR** |  | 0.865 (0.726-1.031) | 0.105 |
| **mSIS** | Score 0: ALB≥ 4.0 g/dL and LMR≥3.4 |  | 0.316 |
|  | Score 1: ALB<4.0 g/dL or LMR<3.4 | 1.579 (0.871-2.860) | 0.132 |
|  | Score 2: ALB<4.0 g/dL and LMR<3.4 | 1.362 (0.607-3.058) | 0.453 |
| **PNI** | PNI= 10×serum albumin (g/dl) + 0.005×total lymphocyte count (per mm^3^) | 0.749 (0.699-0.803) | <0.001 |

NLR, neutrophil-lymphocyte ratio; PLR, platelet-lymphocyte ratio; LMR, lymphocyte-monocyte ratio; ALB, albumin; OR, odds ratio; CI, confidence interval; mSIS, modified systemic inflammation score; PNI, prognostic nutrition index;
