# Supplementary material for: Risk factors for unplanned intensive care unit admission after esophagectomy: a retrospective cohort study of 628 patients with esophageal cancer
Source: Front Oncol. 2024 Aug 29;14:1420446. doi: 10.3389/fonc.2024.1420446 (PMC11390390; doi:10.3389/fonc.2024.1420446)
Supplement: Supplementary file 3 [file Table3.docx]

Supplementary Table 3. Postoperative complications and survival data of the cohorts.

|  |  | **Total population** | | |
| --- | --- | --- | --- | --- |
| **Variable** |  | **UIA (n=57)** | **no UIA (n=571)** | **P value** |
| **Anastomotic leakage^$^** | n (%) | 8 (14%) | 60 (10.5%) | 0.414 |
| **Respiratory system*** | n (%) | 39 (68.4%) | 309 (54.1%) | 0.038 |
| **Cardiovascular system^&^** | n (%) | 33 (57.9%) | 67 (11.7%) | <0.001 |
| **Thromboembolic complication^#^** | n (%) | 17 (29.8%) | 83 (14.5%) | 0.003 |
| **Hospital mortality** | n (%) | 10 (17.5%) | 8 (1.4%) | <0.001 |
| **Length of hospital stay** | mean±SD, day | 31.44±2.84 | 20.63±0.34 | <0.001 |
| **Total medical costs** | mean±SD, CNY | 154178.87±10468.19 | 108097.46±887.25 | <0.001 |
| **Median survival** | median (95% CI), month | 30 (15.4, 44.6) | 48 (36.1, 59.9) | 0.025^ |

CNY, China Yuan; SD, standard deviation; UIA, unplanned intensive care unit admission

^$^including tracheoesophageal fistula

*including pneumonia, atelectasis, hydrothorax, chylothorax and respiratory failure

^&^including cardiac failure, hypotensive shock, hypertensive crisis and arrhythmia

^#^including superficial/deep venous thrombosis, pulmonary embolism, myocardial infarction, and cerebral infarction

^log-rank test
