# Supplementary material for: Risk factors for unplanned intensive care unit admission after esophagectomy: a retrospective cohort study of 628 patients with esophageal cancer
Source: Front Oncol. 2024 Aug 29;14:1420446. doi: 10.3389/fonc.2024.1420446 (PMC11390390; doi:10.3389/fonc.2024.1420446)
Supplement: Supplementary file 4 [file Table4.docx]

**Supplementary Table 4. Subgroup analysis comparing minimally invasive versus hybrid/open procedures.**

|  |  | **Total population** | | |
| --- | --- | --- | --- | --- |
| **Variable** |  | **Minimally invasive (n=521)** | **Hybrid/open# (n=107)** | **P value** |
| **Age, mean±SD** |  | 64.4±0.33 | 64.5±0.81 | 0.846 |
| **Sex, n (%)** | Male | 368 (70.6%) | 81 (75.7%) | 0.290 |
|  | Female | 153 (29.4%) | 26 (24.3%) |  |
| **BMI, mean±SD** |  | 23.6±0.14 | 23.9±0.35 | 0.453 |
| **ASA classification, n (%)** | I/II | 317 (60.8%) | 60 (56.1%) | 0.359 |
|  | III | 204 (39.2%) | 47 (43.9%) |  |
| **CCI, n (%)** | 0 | 253(48.6%) | 57 (53.3%) | 0.290 |
|  | 1 | 194 (37.2%) | 30 (28.0%) |  |
|  | 2 | 57 (10.9%) | 17 (15.9%) |  |
|  | ≥3 | 17 (3.3%) | 3 (2.8%) |  |
| **COPD, n (%)** | Yes | 132(25.3%) | 29(27.1%) | 0.703 |
|  | No | 389 (74.7%) | 78 (72.9%) |  |
| **Tumor location￡, n (%)** | Upper | 69 (13.2%) | 11 (10.3%) | 0.622 |
|  | Middle | 196 (37.6%) | 39 (36.4%) |  |
|  | Lower | 256 (49.1%) | 57 (53.3%) |  |
| **Pathology, n (%)** | ESCC | 460 (88.3%) | 94 (87.9%) | 0.897 |
|  | Others* | 61 (11.7%) | 13 (12.1%) |  |
| **T stage&, n (%)** | T1 | 150 (28.8%) | 23 (21.5%) | 0.098 |
|  | T2 | 165 (31.7%) | 30 (28.0%) |  |
|  | T3/4a | 206 (39.5%) | 54 (50.5%) |  |
| **N stage&, n (%)** | N0 | 309 (59.3%) | 61(57.0%) | 0.660 |
|  | N+ | 212 (40.7%) | 46 (43.0%) |  |
| **Neoadjuvant therapy, n (%)** | Yes | 91(17.5%) | 24 (22.4%) | 0.227 |
|  | No | 430 (82.5%) | 83 (77.6%) |  |
| **Lymphadenectomy, n (%)** | Two-field | 446(85.6%) | 94 (87.9%) | 0.542 |
|  | Three-field | 75 (14.4%) | 13 (12.1%) |  |
| **Intraoperative hypotension, n (%)** | Yes | 91 (17.5%) | 20 (18.7%) | 0.762 |
|  | No | 430 (82.5%) | 87 (81.3%) |  |
| **Intraoperative bradycardia, n (%)** | Yes | 238 (45.7%) | 47 (43.9%) | 0.740 |
|  | No | 283 (54.3%) | 60 (56.1%) |  |
| **Operation time, mean±SD** |  | 308.3±3.08 | 315.6±6.90 | 0.330 |
| **Blood transfusion, n (%)** | Yes | 27 (5.2%) | 16 (15.0%) | 0.001 |
|  | No | 494 (94.8%) | 91 (85.0%) |  |
| **RLNP, n (%)** | Yes | 49 (9.4%) | 9 (8.4%) | 0.746 |
|  | No | 472 (90.6%) | 98 (91.6%) |  |
| **PNI** |  | 53.1±0.24 | 52.6±0.60 | 0.221 |

ASA, American Society of Anesthesiologists; BMI, body mass index; CCI, Charlson Comorbidity Index; COPD, chronic obstructive pulmonary disease; ESCC: esophageal squamous cell carcinoma; N, node; PNI, prognostic nutrition index; RLNP, recurrent laryngeal nerve paralysis; SD, standard deviation; T, tumor; UIA, unplanned intensive care unit admission

*Adenocarcinoma and small cell carcinoma

^#^Including thoracotomy and/or laparotomy

^&^Clinical stage at first diagnosis based on chest CT and ultrasound gastroscopy; stages T3 and T4a were combined in the analysis.

**^￡^**The tumor location was categorized according to the 12th edition of the Japanese Classification of Esophageal Cancer.
