# Supplementary figures and images for: Risk factors for unplanned intensive care unit admission after esophagectomy: a retrospective cohort study of 628 patients with esophageal cancer
Source: Front Oncol. 2024 Aug 29;14:1420446. doi: 10.3389/fonc.2024.1420446 (PMC11390390; doi:10.3389/fonc.2024.1420446)

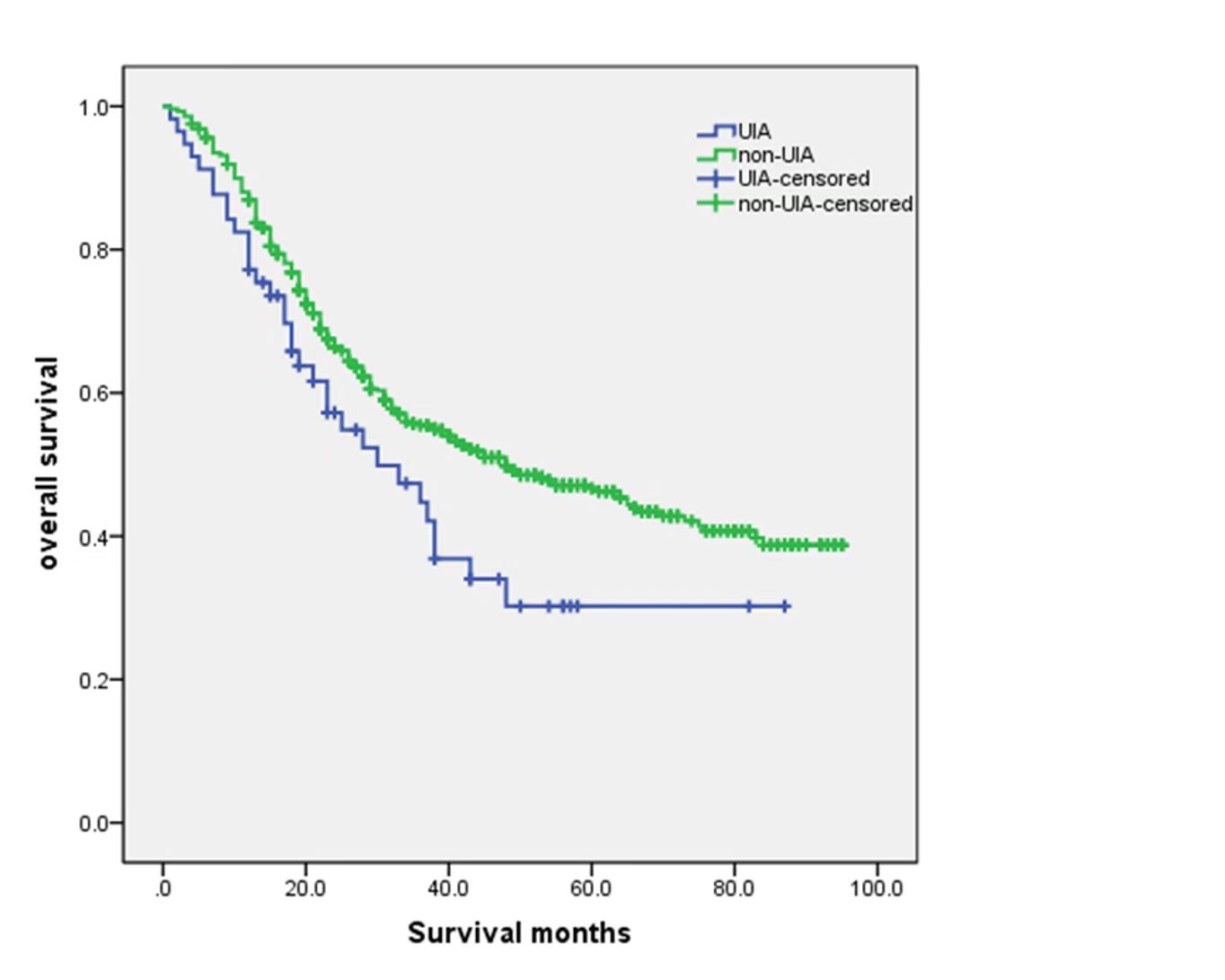

Supplement: Supplementary file 5 [file Image1.jpg]
